# Spindle cell oncocytoma of the pituitary tumor: A rare case report and literature reviews

**DOI:** 10.3389/fsurg.2022.1021680

**Published:** 2023-01-30

**Authors:** Chia Ning Chang, Chiung Chyi Shen

**Affiliations:** Department of Neurosurgery, Neurological Institute, Taichung Veterans General Hospital, Taichung, Taiwan

**Keywords:** pituitary tumor, spindle cell, benigh tumors, TTF-1 gene, surgical resection

## Abstract

**Introduction:**

Spindle cell oncocytoma (SCO) of the pituitary gland is increasingly established with improvements in histological and immunohistochemical examination. However, the diagnosis was often mistaken based on imaging studies and nonspecific clinical manifestations.

**Purpose:**

This case is presented to provide an overview of the characteristics of the rare tumor as well as to demonstrate the difficulties in diagnosis and current treatments.

**Clinical discussion:**

The pathogenesis of SCO remains unclear, and a possible origin was described. Further research is needed to optimize pre-operative diagnosis and surgical strategy.

**Conclusion:**

SCO should be considered when images indicate some features. Gross total resection (GTR) after surgery seems to have better long-term tumor control, and radiotherapy may help decrease tumor progression in patients with non-GTR. Regular follow-up is advised because of the higher recurrence rate.

## Introduction

Spindle cell oncocytoma (SCO) of the adenohypophysis is a rare neoplasm of the pituitary gland first described by Roncaroli et al. (1), and has been included in the World Health Organization (WHO)'s classification of the central nervous system since 2007.

Clinically and radiologically, these neoplasms are often indistinguishable from nonfunctioning pituitary adenomas. It is a challenge for surgeons to evaluate and manage. We presented herein a case of SCO and reviewed the literature, focusing on tumor origin, radiological features, and treatment choice.

## Case presentation

**Figure 1:**
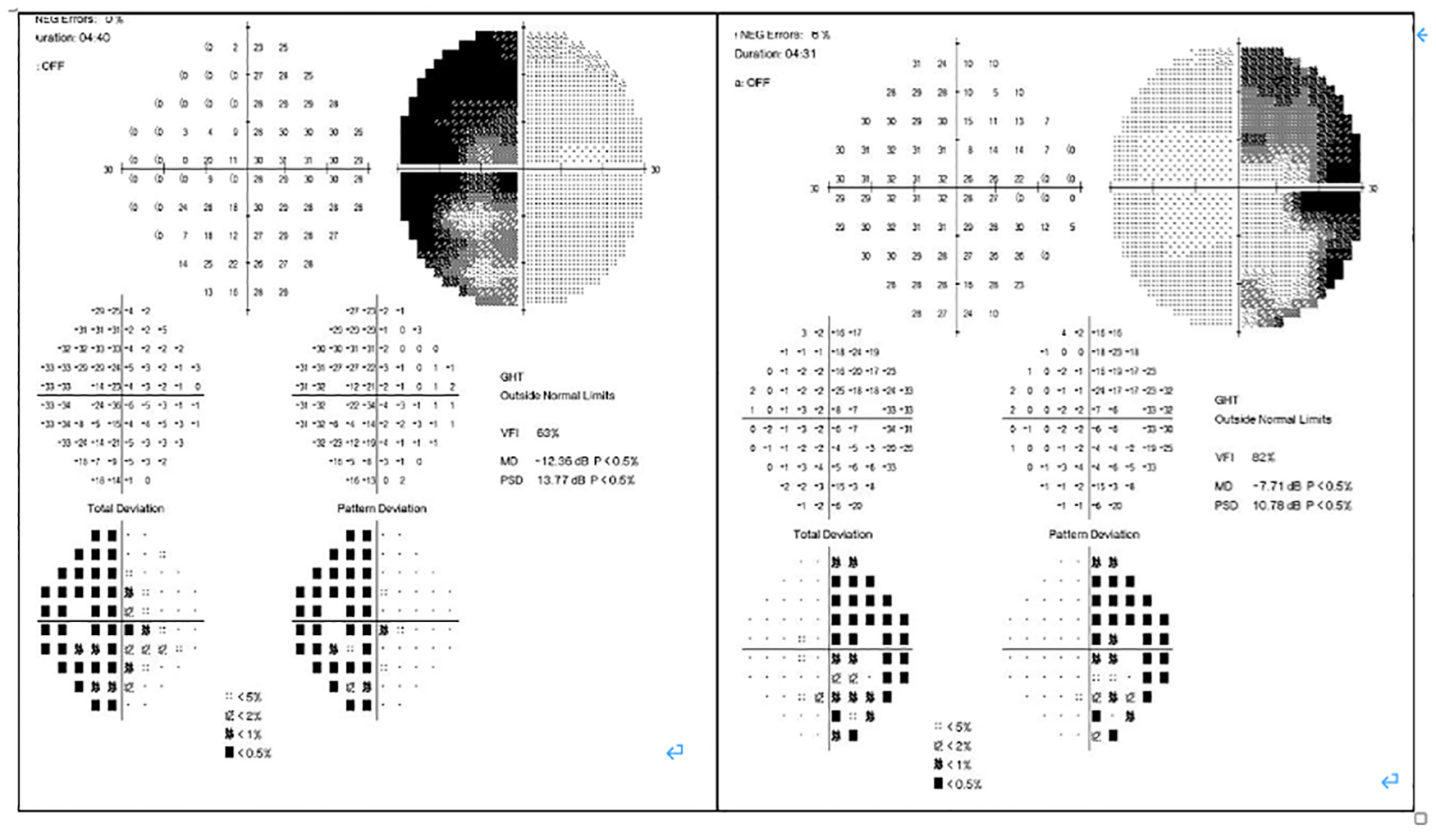
Pre-operative visual field test.

**Figure 2:**
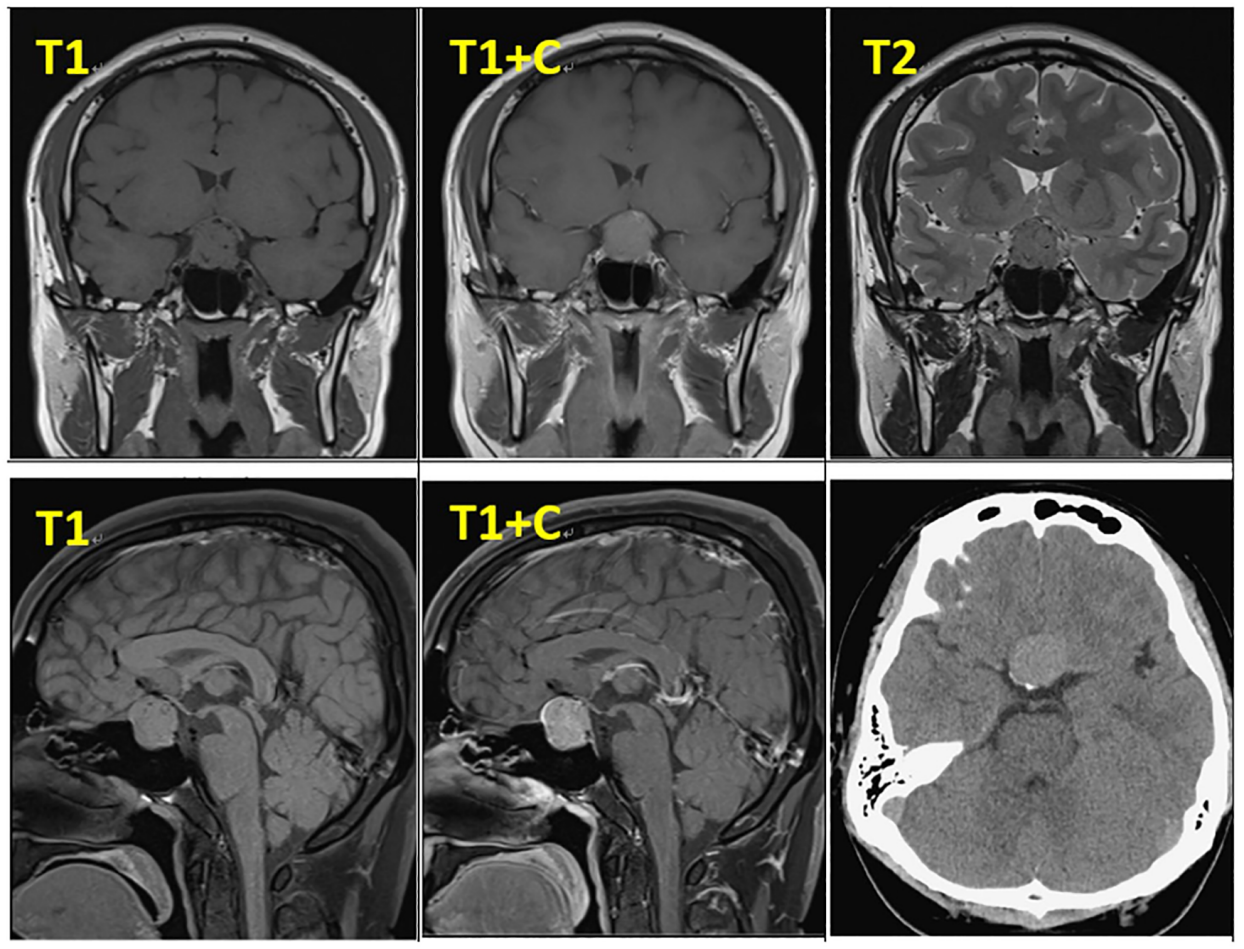
Pre-operative MRI and CT.

**Figure 3:**
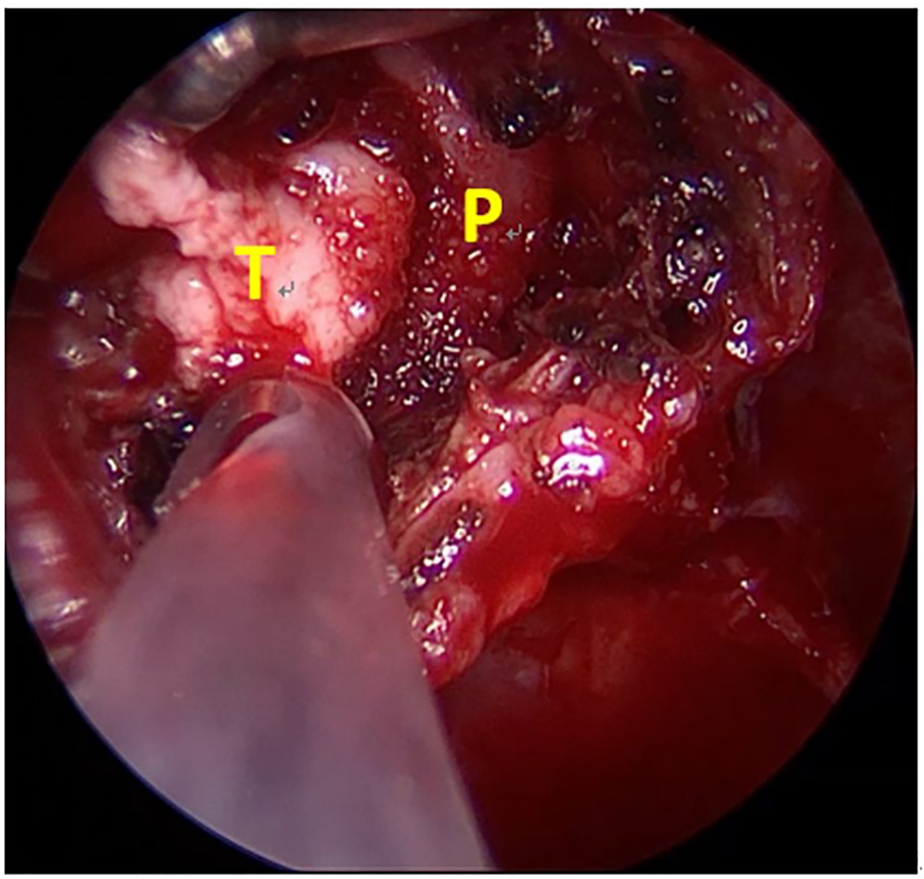
Intraoperative picture. P, normal pituitary gland after tumor removal. T, the gross appearance of the tumor.

**Figure 4:**
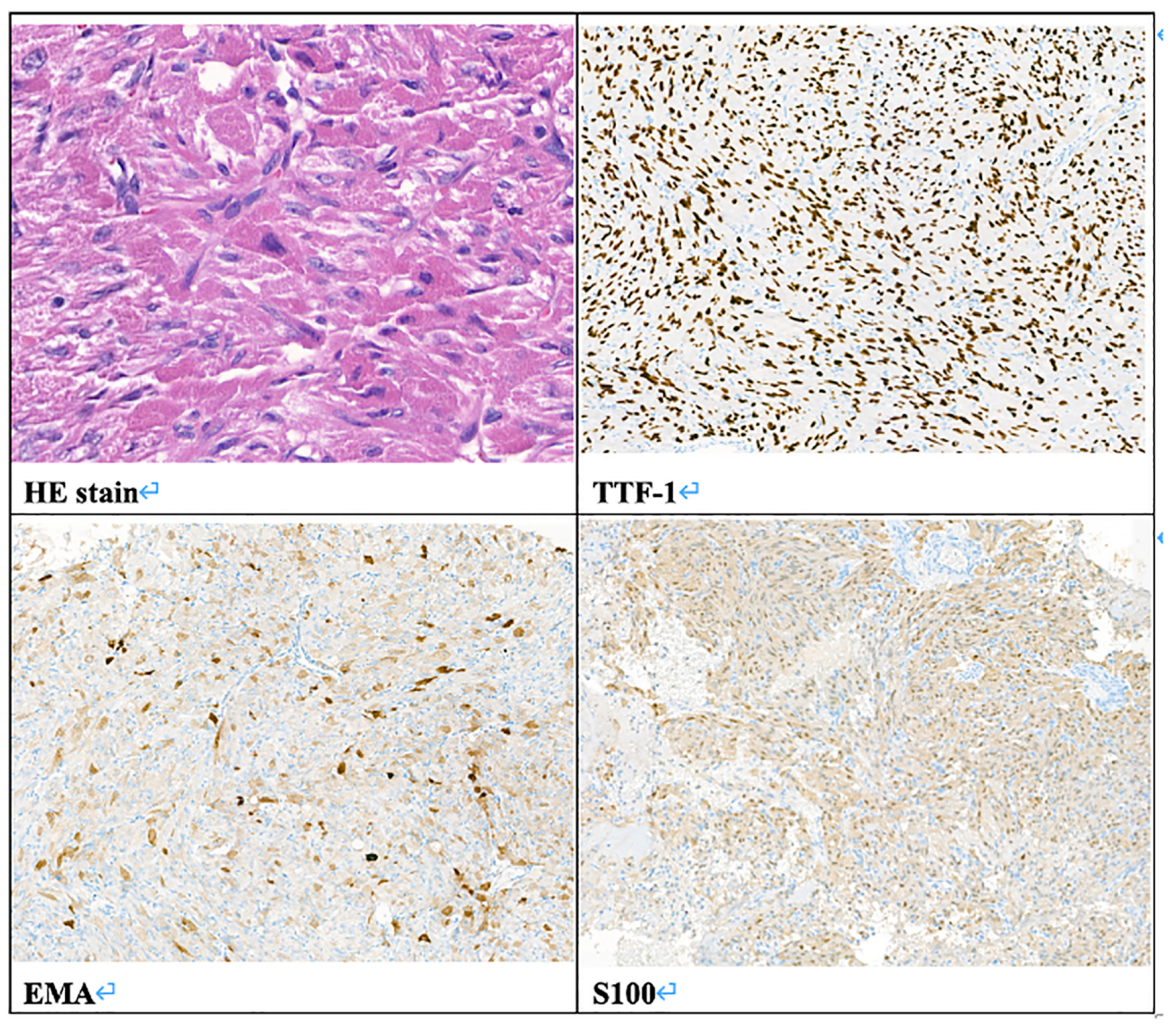
Pathologic report. Histology of the tumor. HE stain, original magnification 400×. Immunohistochemistry stain, TTF-1 (+), EMA (+), S100 (+), original magnification 100×.

**Figure 5:**
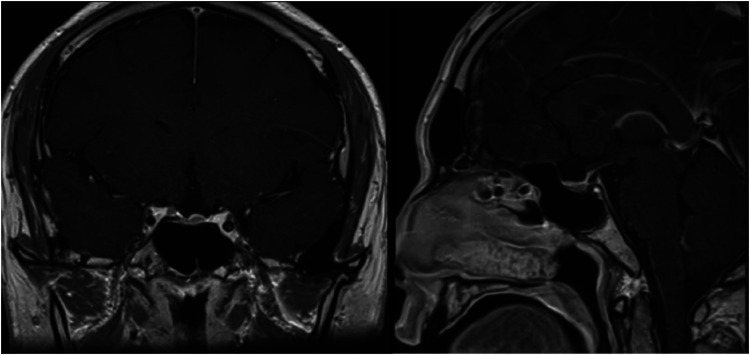
Post-operative MRI after 18 months.

In February 2019, a 31-year-old man without medical history came to our hospital with the chief complaint of progressive bilateral vision loss for 6 months. In the outpatient department, his visual field test (Figure 1) showed a temporal defect in both eyes. Brain computed tomography (Figure 2) showed increased soft tissue density over the sellar and suprasellar regions. Brain magnetic resonance imaging (Figure 2) was further arranged, which showed a mass with enhancement measuring about 2.3 cm × 2.3 cm × 2.0 cm in size located at the sella with suprasellar extension. Under the impression of a pituitary tumor with optic chiasm compression, he was admitted for further evaluation and management. On admission, his neurological examination revealed essentially negative findings, except for loss of visual field; his hormone studies were within normal limits. Under general anesthesia, he underwent an operation using the endoscopic endonasal transsphenoidal approach for the removal of the tumor. Grossly, the tumor was yellow and soft in consistency (Figure 3). Easy bleeding of the tumor was noted and could be controlled under assistance of hemostatic agent. After surgery, his vision was subjectively improved. Histological examination (Figure 4) showed tumor growth exhibiting spindle to epithelioid cells, featuring eosinophilic and granular cytoplasm. Immunohistochemically, the neoplastic cells showed TTF-1 (+), EMA (+), and S-100 (+). Based on the results of the histopathology findings and immunohistochemical stains, SCO was diagnosed. He was discharged in good condition and has been receiving regular follow-up at our neurosurgery department (Figure 5).

## Discussion

SCO is a rare tumor of the adenohypophysis and has been characterized as spindle to epithelioid cells with an oncocytic appearance (1). SCO has similar clinical and radiographic features to nonfunctioning pituitary adenoma and is a challenge to diagnose pre-operatively. To date, fewer than 100 cases of pituitary SCO have been reported in the literature.

Its pathogenesis and natural history have not yet been fully clarified. Nevertheless, there are distinct features of SCO that are becoming increasingly apparent in previously documented case reports.

SCO can be characterized by its ultrastructural/histologic findings. There are typically elongated or polygonal spindle cells with numerous mitochondria with lamellar cristae, small numbers of lysosomes, and rough endoplasmic reticulum (1, 2). These neoplastic cells are linked by intermediate junctions and desmosomes (1). Mitosis is rare, and necrosis is not seen (1).

SCO has no immunoreactivity for neuroendocrine markers and hormones, but shows simultaneous positivity to vimentin, galectin-3, S-100 protein, TTF-1, antimitochondrial antibody MU213-UC clone 131, and frequently EMA. These tumor cells do not express GFAP, cytokeratins CAM2.5, CD34, CD68, synaptophysin, chromogranin, BCL-2, or smooth muscle actin (1, 3). Similar expression of vimentin, S-100 protein, EMA, and galectin-3 together with the presence of desmosomes and intermediate junctions suggests their possible derivation from folliculostellate cells of the adenohypophysis, which are capable of divergent differentiation as a type of stem cell (4, 5).

Recently, reports have described diffuse thyroid transcription factor-1 (TTF-1) expression, a specific marker in neurohypophysis (posterior pituitary gland), in SCO, pituicytoma, and granular cell tumors, indicating a pituicyte lineage (6). A new subtype, primary papillary epithelial tumor of the sella (PPETS), has been defined as a tumor entity with papillary architecture and TTF-1 expression (7).

Evidence suggests circumventricular organs, including the neurohypophysis, organum vasculosum of the lamina terminalis, and median eminence, exhibit stem cell properties, which may differentiate toward PPETS (2). However, folliculostellate cells are negative for TTF-1 (2). The pathogenesis of SCO remains a subject of debate.

Given its oncocytic features, the differential diagnosis for SCO may include pituitary neoplasms capable of oncocytic change (especially gonadotrophinomas, null cell adenomas, and rarely silent corticotroph adenomas) (8), metastases from extrahypophyseal oncocytic tumors (1), paragangliomas, meningiomas with oncocytic change, sellar schwannomas (3), and other posterior pituitary tumors.

Pre-operative symptoms are not diagnostic, and the most common symptoms are the results of the mass effect on parasellar structures. The most clinical complaint is visual impairment (9), as in our case.

**Figure 6:**
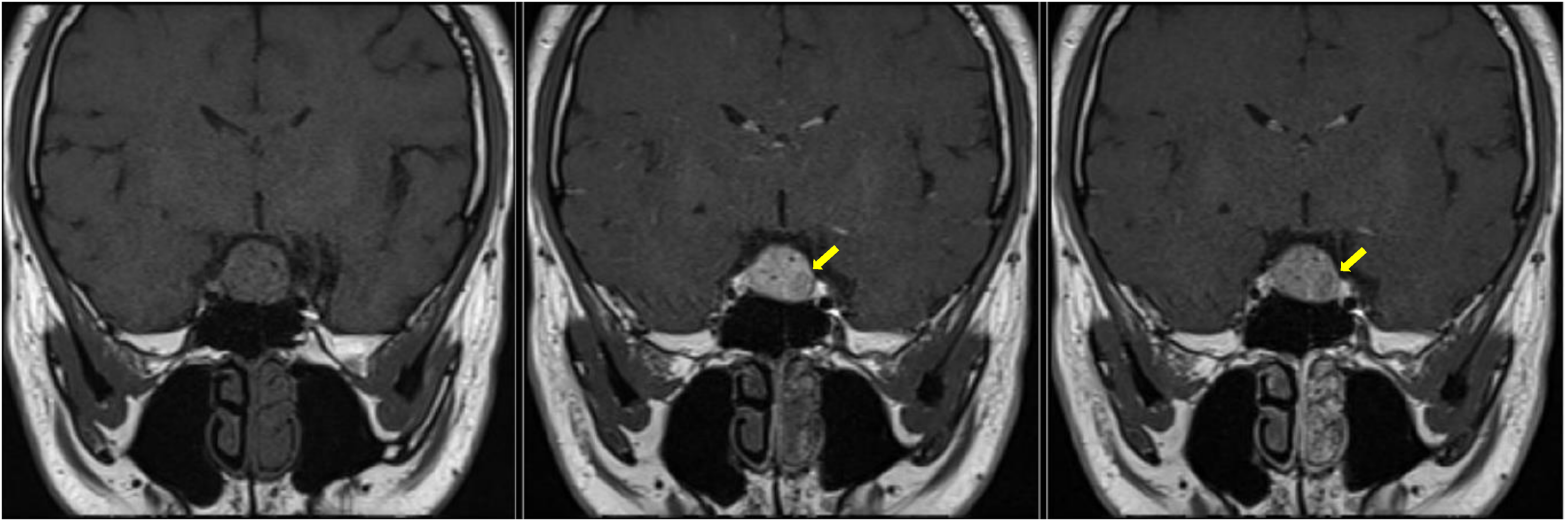
Dynamic contrast MRI (pre-operative). Yellow arrowhead, normal pituitary gland.

Radiologically, there are generally no definitive signs for the diagnosis of SCO, but some radiological clues can be found. SCOs are usually isointense on T1-weighted imaging with flow voids (10). They tend to have a missing posterior pituitary spot, as the posterior pituitary gland cannot be seen separately from the tumor (11). Borges et al. detailed the features of multifocal intratumoral bleeding with the appearance of a blooming artifact (heterogeneous) in the tumor on MRI (12). Hasiloglu's sign described intense contrast enhancement in the early stage of dynamic contrast-enhanced MRI (DCE-MRI) with some hypointense millimetric foci, which is consistent with hypervascularity and non-enhancing hemosiderin deposits (2). As in our case, the tumor exhibits an early wash-in phenomenon with a subsequent plateau (Figure 6), which unlike most pituitary microadenoma (13). Cavernous sinus invasion, sellar floor destruction, and calcification have rarely been reported (9) (Table 1).

**Table 1:** Radiographic features of SCOs.

MRI	T1WI: Iso-hypoisointensity
	T2WI: Iso-relative hyperintensity
	Avid godolinium enhancement
	Hypervascular signs/hypointensity foci/flow voids
	Missing posterior pituitary bright spot
	Intrasellar with suprasellar extension
DCE-MRI	Early intense contrast enhancement

DCE-MRI, dynamic contrast-enhanced MRI; SCO, spindle cell oncocytoma.

Although SCOs are considered benign tumors based on the absence of mitosis and necrosis (2), one case of malignant change with an increasing ki-67 index was reported (14). A review of the literature found SCOs have a significant risk of progression or higher rate of recurrence than other pituitary adenomas, which is associated with greater tumor size at baseline, duration of follow-up, cavernous invasion, and incomplete resection (8). Patients with subtotal resection were associated with a significantly higher risk for recurrence as compared with the gross total resection (15).

The generally accepted treatment of choice is surgical total resection, *via* the transcranial or transsphenoidal approach. Pre-operative embolization followed by combination with the endonasal approach and the pterional approach was presented in one case of SCO in preparation for bleeding (16). In our case, the extended transsphenoidal approach could provide better exposure compared with the standard approach in dealing with hemostasis. Total resection may be difficult to perform if any of the following occurs: intraoperative bleeding, dura adhesion, unclear margin, firm and elastic tissue texture, invasion of adjacent structures. Other managements involving radiotherapy (external beam radiation, proton beam radiation, CyberKnife, and Gamma Knife radiotherapy) tend to be promising solutions for control of tumor progression (17). The effectiveness and timing of adjuvant radiotherapy have not been well established, though it has been successfully used to achieve remission after mean follow-up of 52 months (18). However, tumor recurrence after radiotherapy was also reported and the sensitivity of SCO to radiotherapy was uncertain (9). Therefore, no clear recommendation is suggested and radiation-related complications should be taken into consideration.

For patients with a radiographically confirmed complete resection, long-term surveillance by annual MRI examination for 5 years and biannually thereafter was preferred (8). Although there is no established therapeutic protocol, long-term follow-up is essential.

## Conclusion

SCO is often initially misdiagnosed as a nonfunctional pituitary adenoma due to its rarity. Comprehensive evaluation, including histopathological, immunohistochemical, radiological features, and intraoperative findings are necessary. Regular follow-up is advised as SCO is associated with a higher recurrence rate. Further research is needed to clarify the pathogenesis and risk factors of SCO.

## Data Availability

The original contributions presented in the study are included in the article/Supplementary Material, further inquiries can be directed to the corresponding author.
